# Estimating the ecological water levels of shallow lakes: a case study in Tangxun Lake, China

**DOI:** 10.1038/s41598-020-62454-5

**Published:** 2020-03-27

**Authors:** Wei Yang, Mingxiang Xu, Ruiqing Li, Liping Zhang, Qiuliang Deng

**Affiliations:** 1 Hubei Provincial Water Resources and Hydropower Planning Survey and Design Institute, Wuhan, 430064 China; 2 State Key Laboratory of Water Resources and Hydropower Engineering Science, Wuhan University, Wuhan, 430072 China

**Keywords:** Freshwater ecology, Wetlands ecology, Limnology

## Abstract

Water level management is an effective tool for the ecological restoration of shallow lakes. In this study, we developed an ecologically-based approach to estimate the monthly suitable ecological water levels (EWLs). This approach took both the lake topographic features and aquatic plants’ growth characteristics into account. The aquatic vegetation coverage was used to characterize the degree of the lake ecological restoration. The relationship between water level and vegetation coverage was established. We chose the Tangxun Lake as a testbed, and the recommended lowest EWL was 16.6 m, as the minimum threshold for water level regulations. The results revealed that the predicted vegetation coverage decreased with the rise of water level during the germination period (February and March). To achieve the vegetation coverage goal of 30% and 50%, the lake’s water levels must be lowered to 17.1 m and 16.8 m respectively during germination. The EWLs were recommended to be low in spring and high in summer, which was matched with the natural water level regimes. The proposed approach can provide a reliable reference for water level regulation of shallow lakes especially the lakes with insufficient data.

## Introduction

Lake is an important part of the natural ecosystem and plays a vital role in human’s survival and development. In the past decades, the hydrological regime of lakes has changed significantly in China due to the synergies of human activities and climate change, leading to a series of ecological problems, such as water environment depravation, eutrophication aggravation and lakeside habitat destruction^1–3^. To address these problems, the government has tried numerous measures including strict control of point source pollution and some bioremediation technologies. Additionally, water level manipulation can be considered an effective tool for wetland restoration^4,5^. Water level is an important characteristic index to reflect the lakes’ hydrological regime, as well as a key factor influencing the distribution and diversity of aquatic plants in shallow lakes^6,7^. To maintain the lakes’ basic ecological functions, the concept of lake ecological water level (EWL) was developed. And it was defined as the optimal water level for maintaining the ecosystem integrity, protecting the biodiversity, improving the environmental quality and ensuring the ecosystem stability^8,9^. A thorough understanding of EWL is critical to restoring the lake ecosystems^10^.

There has been an increasing number of studies on the EWL of lakes, and these studies mainly focus on two aspects: (1) the influences of water level fluctuations on the establishment and development of biological communities in lakes; (2) the active or passive responses of biological communities to the water level fluctuations of lakes. Water level fluctuations, especially their extent, frequency and duration, have great effects on the lake’s physical environment and biological communities^6,11^. Particularly, since aquatic plants play a vital role in the maintenance of lake ecosystem health, much research has been undertaken to investigate the impacts of water level fluctuations on aquatic plants by using ecological indicators such as biodiversity and coverage^12–14^. It has been found that minor changes in water levels could produce huge alterations in plant communities^15^. Moreover, the rates of water level change are also dominant forces affecting the growth and development of aquatic plants. Aquatic plants gradually adapt to the natural hydrological regime in the long process of evolution, and they have different water level requirements at different growth stages^16^. Based on this, many countries have attempted to formulate water level manipulation schemes to restore natural water level regimes and ensure the species diversity and ecosystem stability of lake wetlands.

How to determine the appropriate EWLs in shallow lakes has become a new research hotspot. Since the end of the 20th century, a variety of methods have been developed for calculating the EWLs. The scope of the EWLs assessment methods has gradually broadened from the hydrological analyses, towards more comprehensive approaches. Commonly used methodologies fall into four categories: historical flow record methodologies, hydraulic rating methodologies, habitat rating methodologies, and holistic methodologies^17^. The historical flow record methodologies and the hydraulic rating methodologies rely heavily on the availability of reliable long-term hydrological data^9,18^. These two methodologies are based on the concept that water levels below the natural water levels will destroy the ecosystem integrity^19^. The habitat rating methodologies were derived from the hydraulic rating methodologies and were widely used in recent years. They draw on the ecological requirements of indicator species to assess the suitability for ecosystem processes. And the common approaches include the Physical Habitat Simulation Model^20^, the Minimum Biological Space Requirements Method^21^, the Instream Flow Incremental Methodology^22^ and the Habitat Analysis Method^23^. The holistic methodologies are comprehensive multidisciplinary approaches considering both hydrological and ecological indicators. Since the interaction mechanism between hydrological factors and ecological factors is extremely complicated, the research of this methodology is still at its initial period^24^. Among the existing methodologies, most of them were developed to determine the environmental flow requirements of rivers, while they could not directly applicable to lake wetlands. Moreover, the primary focuses of these methods have been on the lowest EWLs, with less emphasis on the effects of dynamic water level fluctuations on the aquatic species. However, a single EWL value could not meet the water level requirements at different growth stages of aquatic species^25,26^. The extent, frequency and duration of water levels are more worthy of attention. Therefore, further studies are still necessary to develop the methods for EWLs calculation in lakes that could meet the water level requirements at different growth stages of aquatic species.

In this study, we developed an approach for calculating the suitable monthly EWLs of shallow lakes. The essence of this approach was to regulate the water level based on the water level requirements of aquatic plants. The vegetation coverage was used as an indicator of ecological health in this approach. The lowest EWL was calculated as the minimum threshold for water level regulation using three approaches. The principle and procedure of this method were introduced in detail, and then the methodology was applied to the Tangxun Lake.

## Materials and Methods

### Study area

**Figure 1:**
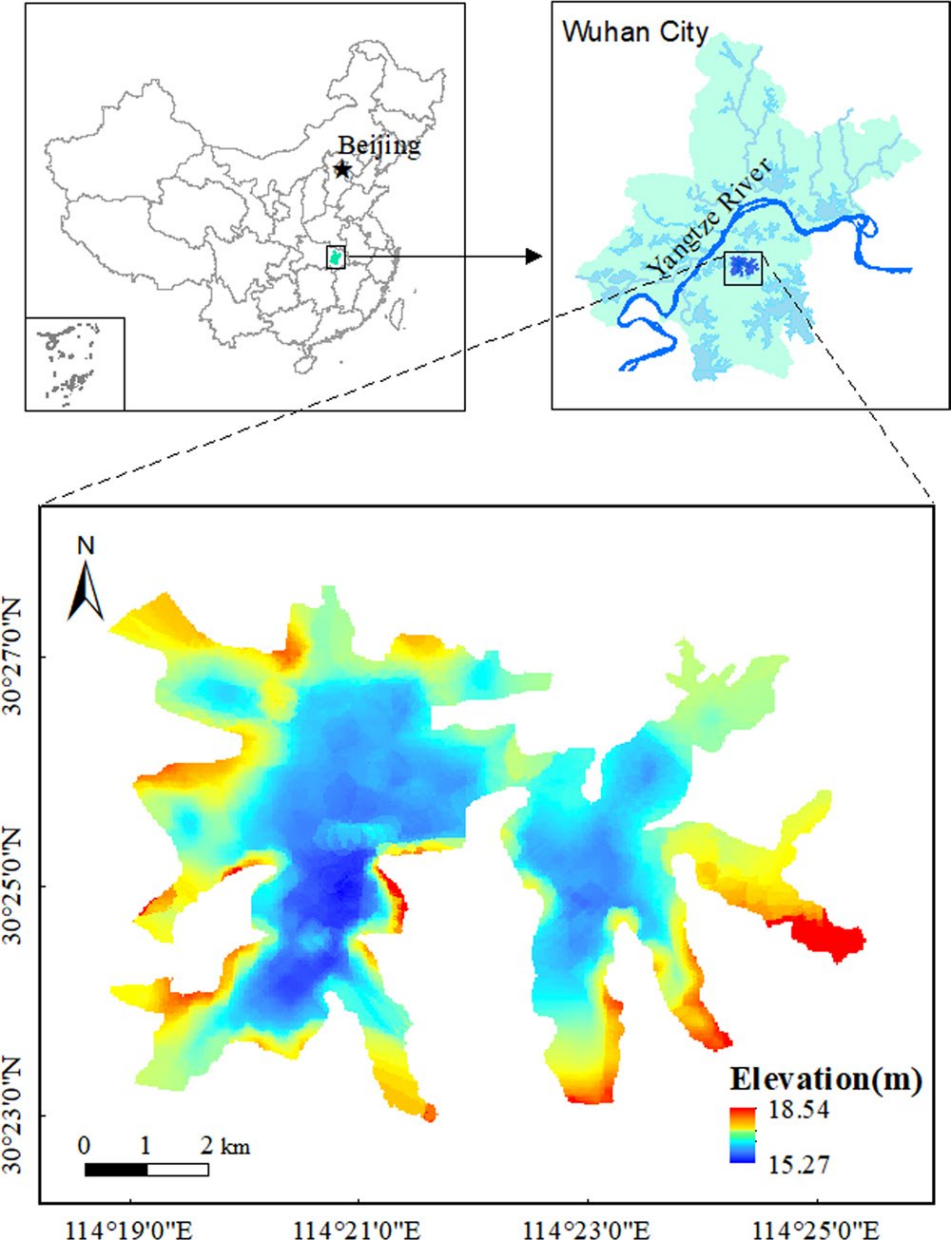
Location sketch of study area and its underwater terrain map.

In this study, we selected the Tangxun Lake in China as the study area. It is the largest urban lake in China and located in the middle and lower reaches of the Yangtze River (Fig. 1). The Tangxun Lake has a water surface area of 52.19 km^2^ and drains a watershed area of 240.38 km^2^. It is a typical subtropics shallow lake with an average water depth of 1.85 m. It serves as the main water source of drinking, irrigation and aquaculture for Wuhan, the capital city of Hubei Province.

The Tangxun Lake used to be the largest original ecological lake in Wuhan. However, in recent years, the increase of population and economic development in the basin, especially the construction of industrial parks and development zones around the basin since 1996, has discharged a large amount of pollutant load that exceeds the environmental capacity of the water body, resulting in water pollution and eutrophication in the lake. As a result, the biological diversity and the aquatic vegetation coverage are sharply reduced, which poses a serious threat to the health of the surrounding residents.

### Traditional methods for estimating EWLs

The lowest EWLs are generally emphasized in traditional methods of estimating the EWLs, and are usually estimated through the following approaches.

#### Lake morphological analysis method (LMAM)

The LMAM was proposed by Xu *et al*. and was widely used in China^27^. In this method, water level is used as the index of lake topography and hydrological condition, and lake area is used as the index of lake function. Based on the measured water level and lake area data, the relation curve between water level and lake area can be established. The change rate of lake area is the first derivative of the relation function between lake area and water level. The water level corresponding to the maximum change rate of the lake area is treated as the lowest EWL of the lake. A major assumption of this method is that if the water level is lower than the lowest ecological water level, the surface area of the lake will be significantly reduced and the lake function will be seriously degraded.

This method can be expressed as:

1 $$F=f(H)$$

2 $$\frac{{\partial }^{2}F}{\partial {H}^{2}}=0$$

where *F* is lake area (m^2^); *H* is water level (m). The lowest EWL can be obtained by solving the equations.

#### Natural water level statistics (NWLS)

Some researchers demonstrated that the annual and inter-annual changes in water level cause disturbance to the lake ecosystem under natural conditions^21^. The premise of the NWLS is that the lake ecosystem has adapted to the disturbance of lake level during the long ecological evolution^28^. The long-term daily water level data is required in this method, then the water level guarantee rate curve can be plotted. The water level with guarantee rate of 95% is generally considered as the lowest EWL^29,30^.

#### Biological living space requirement method (BLSRM)

The aquatic organisms in lakes include phytoplankton, emergent plants, zooplankton, fishes and so on. Each biological community needs a minimum living space to maintain its own community from severe recession. The water level corresponding to this living space is the lowest EWL of the lake. The key issue is to identify the organism most sensitive to the water level and then determine the lowest water level that the organism requires to survive and reproduce.

The lowest EWL can be calculated as:

3 $${H}_{{\rm{\min }}}={H}_{b}+{h}_{c}$$

where *H*_min_ is the lowest EWL of the lake; *H*_*b*_ is the bottom elevation of the lake; *h*_*c*_ is the lowest water depth that the organism requires.

### The proposed approach for estimating the suitable EWLs

#### Fundamental principles of the approach

The aquatic vegetation coverage, which is defined as the percentage of aquatic vegetation area in the total area of the lake, is a very important index to score the growth conditions of aquatic plants in the lake ecosystems. In this approach, it was used as the main ecological restoration target in the regulation of ecological water level.

The growth and reproduction of aquatic vegetation are closely related to the water level fluctuation. The growth periods of aquatic plants in subtropical lakes are generally divided into six stages: germination (February-March), seedling growth (April-May), growth and diffusion (June-July), maturation (August-September), seed propagation (October-November) and dormancy (December-January). Among these growth periods, the germination stage is particularly important since it determines the plant distribution and vegetation coverage of the lakes. Therefore, February and March is regarded as the critical period of aquatic vegetation restoration and the benchmark period of water level regulation. Then the suitable EWLs of the lake can be obtained based on the water depth and water level variation requirements of indicator species.

#### Calculation method framework

**Figure 2:**
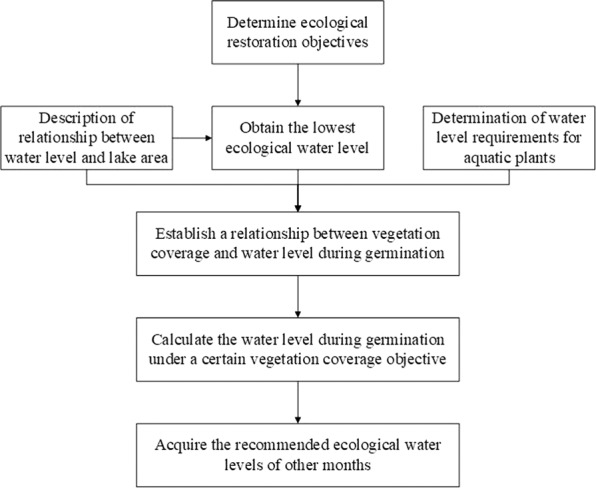
The framework of the proposed approach.

The framework of this approach was shown in Fig. 2. The lowest EWL was considered as the minimum threshold level for maintaining the functional integrity and biodiversity of the lake ecosystems, and it was calculated using the methods mentioned above. Afterwards, the relation curve between water level and its corresponding water area in lakes was plotted by linking water level data with underwater topography data. Furthermore, a specific quantitative relationship between vegetation coverage and water levels during germination was established based on the water requirements for aquatic plants. Based on this, the recommended water levels during germination were derived once the vegetation coverage objectives were determined. Then the suitable EWLs of other months could be estimated according to the requirements of aquatic plants in water-level changing speed and water depth.

#### Water level requirements of aquatic plants

Aquatic plants in lake ecosystems mainly include several types, such as emergent plants, hygrophytes, floating-leaved plants and submerged plants, and most of them are located in the lakeside zone. The emergent plants and hygrophytes can both germinate in shallow areas of the lake, while the floating-leaved plants and submerged plants are usually found under water. Previous research showed that submerged plants could develop only when the ratio of Secchi Depth (SD) to water depth was beyond 0.6, and the emergent plants could develop where the water depth was less than 20 cm^16^. Most of the aquatic vegetation in Tangxun Lake germinates in February and March. During this time, the lake need to keep a low water level to increase the exposed beach area, and the specific calculation process was shown in the following section. The Phragmites communis community is the predominant community and have wide distributions in the Tangxun Lake wetlands. The growth rate of the Phragmites communis was about 0.7 m/month, and the mean height was 0.6 m in April, 1.0 m in May, 2.2 m in June, and 2.8 m in July, respectively, and stopped growing after August^31,32^. To ensure the normal growth and development of the aquatic plants, the water level must not exceed the tops of the Phragmites communis.

In the seedling growth period (April and May), it is necessary to keep the water level rising steadily and slowly, and the rate of increase should be controlled within 0.6 m/month (Table 1). In the growth and diffusion period (June and July), the lake is suitable to maintain a high water level, but the rising speed should not exceed 0.7 m/month. Maintaining a high water level can not only promote the spread of aquatic plants, but also prevent the lakeside from shrinking. To prevent terrestrial plant invasion and lake swamping, the lake water level is better to keep a high value during the maturation period (August and September), but it should not exceed the warning water level of the lake. During the seed propagation period (October and November), the lake is suitable to maintain a moderate water level. To promote the maturation and spread of seeds, it is necessary to keep the water level steady and slowly decline, and the rate of decline should not exceed 3 cm/day. During the dormancy period (December and January), the water level should maintain a medium or low value.

**Table 1:** Water level requirements of aquatic vegetation during each growth period.

Months	Growth stages	Water level requirements
February-March	Germination	Low water level, higher than the lowest EWL
April-May	Seedling growth	Gradually increased water level, the rising speed must be less than 0.6 m/month
June-July	Growth and Diffusion	High water level, the rising speed must be less than 0.7 m/month
August-September	Maturation	High water level, lower than the warning water level
October-November	Seed propagation	Gradually decreased water level, the drawdown speed must be less than 3 cm/day
December-January	Dormancy	Low water level

#### Calculation steps

The distribution of aquatic plants in a wetland is primarily a function of water depth^14,33^, and the coverage can be calculated according to the water level during the germination period, and the calculation steps are as follows:

**Step 1:** Establish a relationship between water level (*Z*) and lake surface area (*A*).

A series of water surface areas (*A*) corresponding to water levels (*Z*) can be derived from the underwater terrain data of the lake, and then the function relation between *Z* and *A* can be obtained and expressed as *A* = *f*(*Z*).

**Step 2:** Calculate the aquatic vegetation coverage of the lake.

The exposed beach area from February to March was used as the germination and growth area of hygrophytes and emergent plants, and that can be calculated as the surface area between the normal water level (*Z*_*c*_) and the water level during germination (*Z*_*g*_). In addition, previous research showed that the emergent plants could also develop where the water depth was less than 20 cm. Therefore, the elevation distribution of hygrophytes and emergent species was from (*Z*_*g*_ − 0.2 m) to *Z*_*c*_.

It was found that the submerged plants could develop only when the ratio of Secchi Depth (SD) to water depth was beyond 0.6, so the lowest elevation of submerged plants was calculated as *Z*_*g*_ − SD/0.6. The elevation distribution of submerged plants was from (*Z*_*g*_ − SD/0.6) to *Z*_*g*_, which was partially overlapped with hygrophytes and emergent plants.

Therefore, the elevation distribution of submerged plants, hygrophytes and emergent species was (*Z*_*g*_ − SD/0.6)~*Z*_*g*_, *Z*_*g*_ ~ *Z*_*c*_ and (*Z*_*g*_ − 0.2 m) ~ *Z*_*c*_, respectively. The lowest elevation of aquatic plants was the minimum value of *Z*_*g*_ − SD/0.6 and *Z*_*g*_ − 0.2 m, and the elevation distribution of aquatic plants was from min((*Z*_*g*_ − SD/0.6), (*Z*_*g*_ − 0.2 m)) to *Z*_*c*_. The corresponding planimetric area, that was the germination area of aquatic plants, can be calculated according to the function *A* = *f*(*Z*). Hence the coverage of the lake was calculated as:

4 $$\begin{array}{rcl}C & = & \frac{{A}_{c}-\,\min (A\left({Z}_{g}-\frac{SD}{0.6}\right),A({Z}_{g}-0.2))}{{A}_{c}}\times 100 \% \\  & = & \,\frac{f({Z}_{c})-\,\min (f\left({Z}_{g}-\frac{SD}{0.6}\right),f({Z}_{g}-0.2))}{f({Z}_{c})}\times 100 \% \end{array}$$

**Step 3:** Establish a relationship between vegetation coverage (*C*) and water level during germination (*Z*).

Repeating the above calculating steps, the coverage at any germination water level could be calculated. *Z*_*g*_ was assigned a value from *Z*_min_ to *Z*_*c*_, in increments of 0.1 m, where *Z*_min_ was the lowest EWL of the lake, and it can be calculated according to the method mentioned above. Then the relationship between vegetation coverage and water level (*F*_*C~Z*_) can be established.

**Step 4:** Calculate the water level during germination under a given coverage target.

To determine the suitable EWLs under target coverage, the water level at the germination stage should be calculated first. The restoration target of the aquatic vegetation coverage depends on the management goal of the lake. Once the aquatic vegetation coverage is determined, the water level requirement during germination can be calculated using the function *F*_*C~Z*_.

**Step 5:** Determine the suitable EWLs for other growth stages.

Three objectives must be taken into account in the water level manipulation. The first was to rehabilitate the aquatic vegetation coverage. A lowered water level may provide more suitable conditions for germination and seedling growth of hygrophytes and emergent species. Actually, previous studies indicated that the trends of increasing coverage corresponded with low water levels, and decreasing coverage corresponded with high water level^34^. Hence, water level must keep low in spring, but not lower than the lowest EWL. The second was to ensure the normal growth and development of aquatic plants. To achieve this objective, water level requirements of aquatic vegetation in each growth periods should be taken into account. The third was to guarantee the safety of the lakes during flood season. In summer, high water levels were favourable for the dispersal of aquatic plant seeds, and they could also limit exotic vegetation encroachment. However, to guarantee the safety of the lakes, water level should not higher than the warning water level. Based on the above objectives, the upper and lower limits of the water levels can be obtained.

## Results

### Calculation of the lowest EWLs

#### The lowest EWL calculated by LMAM

**Figure 3:**
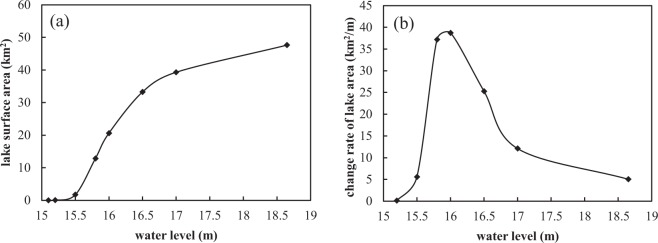
Relationships between water level and (**a**) lake area and (**b**) change rate of lake area.

According to the underwater topography data of the Tangxun Lake, the relationship curve between water level (*Z*) and lake surface area (*A*) was plotted, as shown in Fig. 3(a). Then a series of the change rate of the lake surface area (dF/dZ) corresponding to water levels (*Z*) was derived, and the relation graph was displayed in Fig. 3(b). In this graph, the change rate of the lake surface area reached the maximum value when the water level was 15.95 m, so the lake’s lowest EWL calculated by LMAM was 15.95 m.

#### The lowest EWL calculated by NWLS

**Figure 4:**
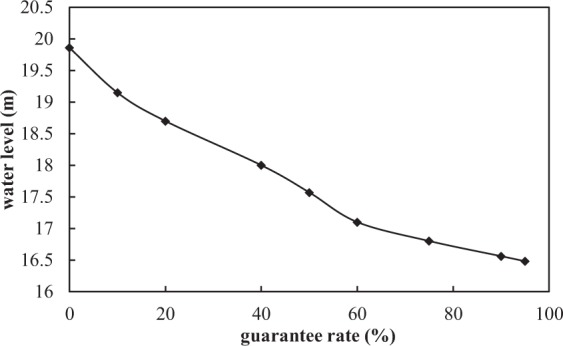
The water level guarantee rate curve. The water level guarantee rate represents the probability that the water level is greater than a certain value.

This paper selected the measured water levels of the lake from 1952 to 1987, which were less affected by artificial regulation and approximate to the natural water level process. The water levels under different guarantee rates can be obtained by arranging the water level data from 1952 to 1987 in the order from small to large, as shown in Fig. 4. It can be discerned from the chart that the water level with guarantee rate of 95% was 16.48 m, so the lake’s lowest EWL calculated by NWLS was 16.48 m.

#### The lowest EWL calculated by BLSRM

Fish has a unique position in the aquatic ecosystem and is one of the top communities in the aquatic ecosystem, which plays an important role in the existence and abundance of other groups. Compared with other biological groups, fish is more sensitive to low water levels, thus fish is selected as the indicator species. Previous studies have indicated that the minimum water depths required for lacustrine fishes to survive are about 1.0 m. According to the underwater topography data, the bottom elevation of the Tangxun Lake was 15.6 m. Therefore, the lowest EWL calculated by BLSRM was 15.6 + 1.0 = 16.6 m.

By using LMAM, NWLS and BLSRM, the lowest EWL was calculated as 15.95 m, 16.48 m and 16.6 m respectively. For safety considerations, the lowest EWL of the Tangxun Lake was recommended as 16.6 m.

### Calculation of the suitable EWLs

Before 2000, the aquatic vegetation coverage of the Tangxun Lake was more than 30%, while after 2013, it was decreased to 10% due to the degradation of the aquatic plants especially the submerged plants. In this paper, the target vegetation coverage was set to be 30~50%.

**Figure 5:**
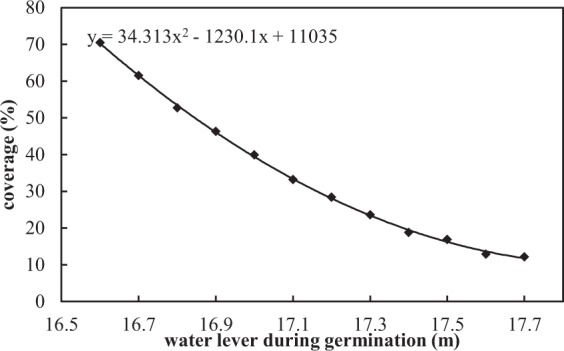
Relationship between vegetation coverage and water level during germination in Tangxun Lake. Points represent the vegetation coverage at each germination water level. Line represents the fitted curves of the relation between vegetation coverage and germination water level.

**Figure 6:**
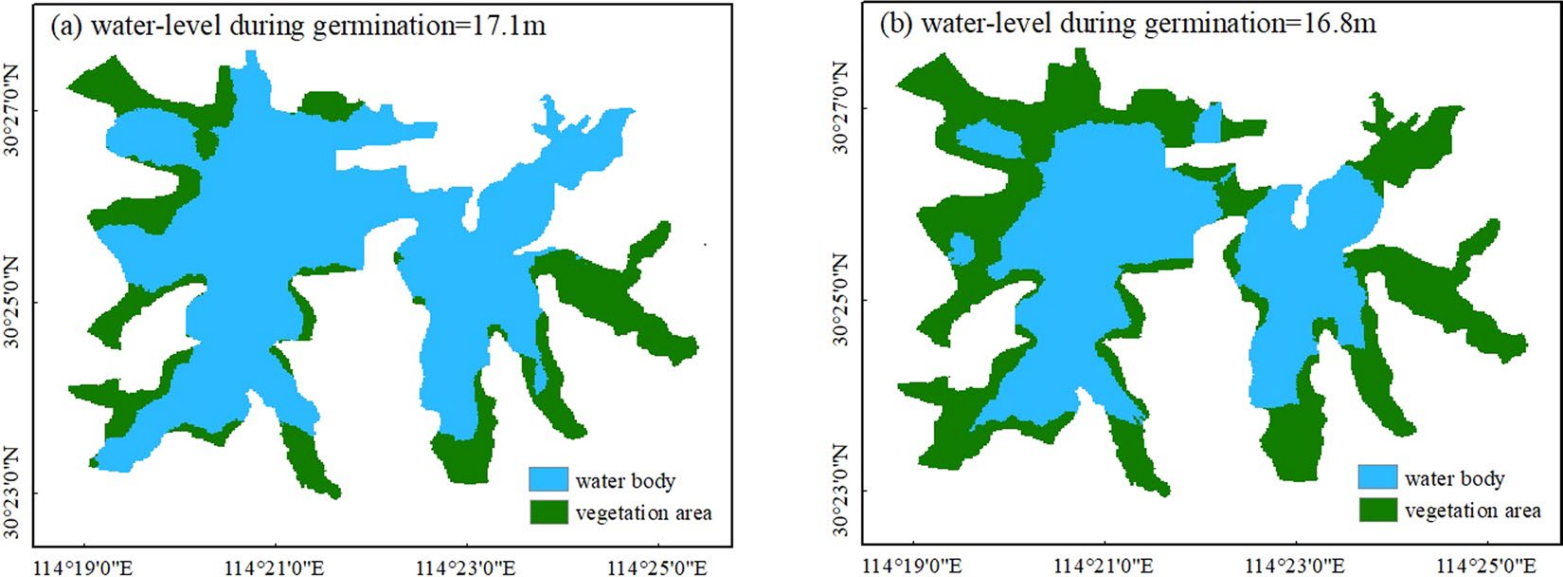
The predicted germination zones of aquatic plants in lakes with the water level of (**a**) 17.1 m and (**b**) 16.8 m during germination.

According to the measured data, the mean Secchi depth (SD) in February and March was about 50 cm, and the normal water level (*Z*_*c*_) was 17.63 m. The lowest EWL of the Tangxun Lake was 16.6 m based on the calculation results in the previous section, so first we assigned the water level during germination period (*Z*_*g*_) to 16.6 m. In this case, the elevation distribution of submerged plants, hygrophytes and emergent species was 15.8~16.6 m, 16.6~17.63 m and 16.4~17.63 m, respectively. Then the coverage (*C*) of the lake was calculated to be 70.5% by the Eq. (4), which means the coverage of the lake could reach to 70.5% when the water level during germination period was 16.6 m. Repeating the above calculating steps, *Z*_*g*_ was assigned a value from 16.6 m to 17.63 m (16.6 m, 16.7 m, …, 17.7 m), in increments of 0.1 m, and the coverage at each germination water level can be calculated, as shown in Fig. 5. The graph indicated that the vegetation coverage decreased with the germination water level rose. Hence a model to predict the coverage of the lake at any potential germination water level was developed by a polynomial fitting method, as expressed to: *C* = 34.313*Z*^2^ − 1230.1*Z* + 11035 (16.6 ≤ Z ≤ 17.7), where *C* was the coverage (%) and *Z* was the water level during germination period (m). Based on this model, the water levels required during the germination stage (February and March) were about 17.1 m and 16.8 m respectively under the coverage scenarios of 30% and 50%. And the predicted germination zones of aquatic plants in lakes were located in the area with low water depth (Fig. 6).

**Figure 7:**
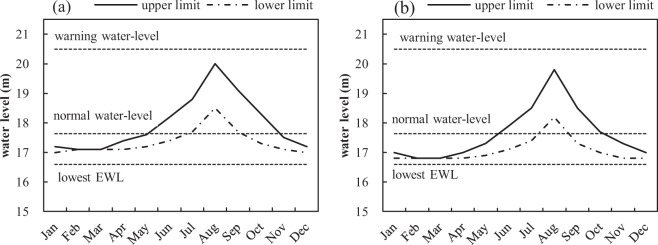
The recommended monthly EWLs to achieve vegetation coverage of (**a**) 30% and (**b**) 50%. The solid lines represent the upper limits of the EWLs, and the dotted lines represent the lower limits of the EWLs.

Based on the water level requirements of aquatic vegetation (Table 1), the recommended suitable EWLs during each growth period can be obtained (Fig. 7). It can be seen from the chart that the water levels fluctuated between the lowest EWL (16.6 m) and the warning water-level (20.5 m). A similar variation trend of the suitable EWLs was observed under the coverage scenarios of 30% and 50%. To achieve the vegetation coverage of 30%, the lake’s water levels remained steady at 17.1 m during germination period (February and March). In the seedling growth period (April and May) and growth diffusion period (June and July), the water level rose slowly, and reached the top in August. There was a gradual decline during the seed propagation period (October and November) and the dormancy period (December and January).

## Discussion and Conclusion

### The relationship between vegetation coverage and water level

Although water level management is an effective tool for the ecological restoration of shallow lakes, the relationship between ecosystem health and water level have rarely been quantified. In this paper, we used the aquatic vegetation coverage to indicate the health of the ecosystem, and then established the quantitative relationship between vegetation coverage (*C*) and water level during germination (*Z*) in the Tangxun Lake, which was expressed as *C* = 34.313*Z*^2^ − 1230.1*Z* + 11035 (16.6 ≤ *Z* ≤ 17.7). The results showed that vegetation coverage decreased as the germination water level increased. The empirical relationship can be used to predict the potential aquatic vegetation coverage under a given water level condition and to help define restoration targets. To maintain the vegetation coverage at 30%, the water level of the Tangxun Lake during germination period must be lowered to 17.1 m. To achieve the vegetation coverage goal of 50%, the water level during the germination period must be lowered to 16.8 m.

**Figure 8:**
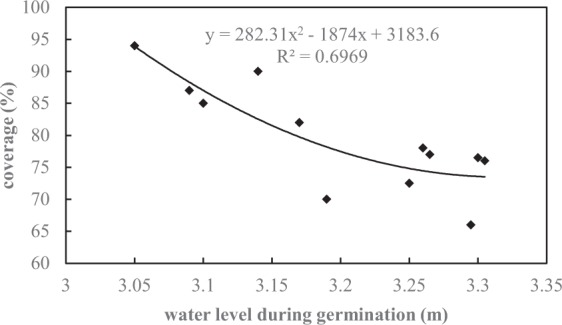
Relationship between vegetation coverage and water level during germination in Eastern Taihu Lake.

Therefore, lowering water level during germination (February and March) is an effective measure for aquatic vegetation restoration. To further confirm this conclusion, we collected the water level and aquatic vegetation coverage of Eastern Taihu Lake from 2003 to 2017^35^ and established a relationship between these two variables, as shown in the Fig. 8. It can be seen that aquatic vegetation coverage and water levels during germination in the Eastern Taihu Lake also presented a negative relationship. In addition, many previous experiment and field studies have shown a notable negative correlation between aquatic vegetation coverage and water level in spring^35–37^. For example, Wu^36^ conducted the experiments with submerged macrophytes in different water levels, and the results showed that high water level in spring was not conducive to the germination of submerged plants due to insufficient underwater light. Therefore, we strongly recommend that the water level of lakes be appropriately lowered in spring on the premise of ensuring the stable basic functions so as to facilitate the germination of aquatic plants.

### Advantages of the proposed approach for estimating the suitable EWLs

It is increasingly recognized that water level management is an effective tool for the ecological restoration of shallow lakes. To determine the specific and feasible management measures for the preservation and restoration of aquatic vegetation, a reliable and flexible method for estimating the EWLs need to be put forward. However, most of the traditional methods focus on the lowest EWL and ignore the effects of water-level fluctuation on aquatic organisms. In this paper, we proposed a simple methodology based on the water level requirement of aquatic plants, which helped stakeholders to determine the lake levels for each period of the year. Depending on the shoreline slope, low water levels in spring can provide suitable conditions for downslope germination and seedling growth of hygrophytes and emergent species^38^, while high water levels in summer can promote the spread of aquatic plants, as well as prevent terrestrial plant invasion and lake swamping. This approach took into account the lake topographic features, as well as the aquatic plants’ growth characteristics. One key aspect of this method was to use vegetation coverage to represent the lake ecosystem health. By means of a response relation analysis between vegetation coverage and germination water level, the vegetation coverage and germination zones of aquatic plants could be predicted once the germination water level has been determined.

The advantage of this methodology for estimating the EWLs is that it is cost-effective and utilises existing data that are available for most shallow lakes. The underwater terrain data of the lake is required in this method, and it can be obtained by field monitoring and remote sensing images^39–41^. It should be noted that the recommendations for EWLs are made under the assumption that vegetation coverage is a good indicator for evaluating the health of lake ecosystems. This assumption relies heavily on the prior knowledge about the water level requirements of aquatic plants. However, due to the complexity of ecosystems, the quantitative relationship between water levels and aquatic plants is not yet completely clear^42,43^. Further knowledge about the water level requirements of aquatic plants is still required to enable more accurate predictions regarding the vegetation coverage in shallow lakes.

Although this article only mentioned aquatic plants, the method can also be extended to other aquatic species, and it depends on the ecological restoration goals of the lake ecosystem. The recommended EWLs can be determined if the water level requirements of aquatic species are available. The methodology developed in this paper is simple and can be conducted over a short time frame. It can provide a reliable reference for water level regulation of shallow lakes especially the lakes with insufficient data.
